# Effects of Sex on the Relationship Between Apolipoprotein E Gene and Serum Lipid Profiles in Alzheimer’s Disease

**DOI:** 10.3389/fnagi.2022.844066

**Published:** 2022-05-30

**Authors:** Jiajia Fu, Yan Huang, Ting Bao, Ruwei Ou, Qianqian Wei, Yongping Chen, Jing Yang, Xueping Chen, Huifang Shang

**Affiliations:** ^1^Laboratory of Neurodegenerative Disorders, National Clinical Research Center for Geriatrics, Department of Neurology, West China Hospital, Sichuan University, Chengdu, China; ^2^Health Management Center, West China Hospital, Sichuan University, Chengdu, China

**Keywords:** Alzheimer’s disease, lipid profiles, sex, *APOE*ε*4*, *APOE*ε*2*

## Abstract

**Background:**

Sex is an important factor in studying the relationship between the *APOE* gene, lipid profiles, and AD. However, few studies have focused on the effect of sex on lipids in AD and normal controls with different *APOE* genes.

**Materials and Methods:**

A total of 549 participants, including 298 AD patients and 251 body mass index (BMI)-matched healthy controls (HCs), were enrolled. Lipid profiles and *APOE* genes in both AD patients and HCs were determined.

**Results:**

(1) TC and LDL were higher in AD patients than in HCs, only in *APOE*ε*4* carrying populations, but not in non-carrying populations. (2) TC and LDL were higher in *APOE*ε*4* allele carriers than in non-carriers, only in AD populations, but not in HCs. (3) The TC of *APOE*ε*2* carriers was lower than that of non-carriers in the male AD population, but not in the female AD population, female HCs, and male HCs. (4) The increased LDL level may increase the risk of AD in female people carrying *APOE*ε*4*.

**Conclusion:**

The TC and LDL levels of *APOE*ε*4* carriers were higher than those of non-carriers, and the effect was more significant in the female AD population. The TC levels in *APOE*ε*2* carriers were lower than those in non-carriers, which was more significant in the male AD population.

## Introduction

Alzheimer’s disease (AD) is the most common neurodegenerative dementia disease which is characterized by insidious onset, progressive memory failure, cognitive impairment, and behavioral and psychological manifestations. The etiology and pathogenesis of AD are still unclear, and the development of AD could be the result of interaction between multiple genetic and environmental risk factors (Poirier et al., 2014). The most specific genetic risk factor for late-onset AD is the Apolipoprotein (*APO*) *E* gene (Corder et al., 1993). The ε2, ε3, and ε4 alleles of the *APOE* gene, located on chromosome 19q13.2, constitute a common polymorphism in most populations (Corder et al., 1993). The *APOE*ε*4* allele is shown to be associated with a higher risk of AD and greater disease severity, whereas the *APOE*ε*2* allele has an opposite role (Corder et al., 1993). Patients with AD have a higher frequency of *APOE*ε*4* allele than control participants (Isbir et al., 2001). An epidemiology study showed that the frequency of the *APOE*ε*4* allele varied drastically among different populations; it occurs in about 25% African Americans, 15% Caucasians, and 7% Chinese (Corbo and Scacchi, 1999).

There are plenty of studies focusing on the correlation between dyslipidemia and AD, but most of them are trying to explore the effect of cholesterol (TC) on AD. A previous study has shown that high serum TC level in middle age is a risk factor for AD and AD-related pathology (Anstey et al., 2008). Cerebrovascular risk factors, such as high cholesterol, had a mild combined effect on the earlier onset of AD (de Oliveira et al., 2014). High TC level in the brain was proven to play an important role in the process of amyloid-β (Aβ)-induced AD (Refolo et al., 2000). TC and low-density lipoprotein (LDL) were shown to be involved in the pathogenesis of AD by increasing amyloid accumulation and disrupting the cell cycle (Wu et al., 2019), but late-life hypercholesterolemia might also slow cognitive decline, particularly when in combination with other cerebrovascular risk factors, possibly due to enhanced cerebral perfusion (de Oliveira et al., 2015). However, a longitudinal study did not show significant associations of high cholesterol with cognitive or functional changes in AD (de Oliveira et al., 2018). Some studies have found that serum HDL levels were lower in AD patients (Merched et al., 2000), and were inversely associated with cognitive impairment, but opposite reports also existed (Launer et al., 2001).

The lipid profiles were also found to be associated with the *APOE*, but the results were inconsistent (Isbir et al., 2001; Hoshino et al., 2002). Previous studies found that the levels of LDL and TC in *APOE*ε*4* carriers increased or tended to be increased compared with non-carriers (Sun et al., 2014). However, Isbir et al. (2001) showed a decreasing trend, and Hall et al. (2006) found no statistical differences. It was also reported that AD patients carrying the *APOE*ε*2* allele had lower TC and LDL levels and higher HDL levels than AD patients carrying the *APOE*ε*4* allele (Sabbagh et al., 2006), but other studies did not find statistical significance (Isbir et al., 2001). de Oliveira et al. (2017) considered that *APOE*ε*4 non*-carriers might enhance lipid availability to protect neuronal membranes, thus overcoming their supposed dysfunction in cholesterol metabolism, while *APOE*ε*4* carriers have inefficient neural repair mechanisms.

In addition, sex can affect *APOE*ε*4* allele-associated cognitive impairment. The risk of AD or MCI conversion was higher in female *APOE*ε*4* allele carriers than that in male *APOE*ε*4* allele carriers (Kim et al., 2015). There was a stronger correlation between *APOE*ε*4* and CSF Tau levels in women than in men (Liu et al., 2021). The gender-specific *APOE* haplotype interactions can alter the response to anticholinesterase therapy (MacGowan et al., 1998). Among the females treated with anticholinesterase, the individuals carrying the *APOE*ε*4* allele presented a poor response to treatment than those carrying other *APOE* alleles, and the anticholinesterase reactivity in the males was superior to that in the females (MacGowan et al., 1998). Some studies found that TC was significantly higher in women with AD (Oliveira et al., 2016). However, few studies have focused on the effect of sex on lipids in AD and normal controls with different *APOE* genes.

Therefore, sex is an important factor affecting the interaction between the *APOE* gene and lipid profiles in AD. The sex-related difference is also crucial for precision therapy. Few studies have clearly elucidated the effect of sex on lipids in AD and normal controls with different *APOE* genes. In order to explore the relationship between lipid profiles, *APOE* gene, and sex in AD, we examined the lipid profiles in AD patients and healthy controls (HCs) with different *APOE* alleles and analyzed the effect of sex on lipid profiles in both AD patients and HCs with different *APOE* genes.

## Materials and Methods

### Participants

Alzheimer’s disease patients admitted to West China Hospital of Sichuan University from January 2020 to January 2021 were recruited, and trained doctors diagnosed AD according to the NINCDS-ADRDA and DSM V (McKhann et al., 1984; dermann and Fleischhacker, 2016). Detailed medical history-taking and physical examination were performed. Individuals without any disease in the central nervous system and normal cognitive function were recruited as healthy controls (HCs) during the same period, and they were matched for body mass index (BMI) to the AD group. The patients received the standardized assessments, including the Mini-mental State Examination (MMSE), the Montreal Cognitive Assessment (MoCA), and magnetic resonance imaging (MRI). AD patients with MMSE scores higher than 25 were excluded. Since MMSE has shown not to be adequate in detecting MCI and clinical signs of dementia, and MoCA is superior to MMSE in identifying MCI (Pinto et al., 2019), HCs with MoCA scores higher than 22 were included in the present study. All participants with vascular dementia (VaD), cardiopathy, hypertension, diabetes mellitus, demyelinating diseases, white matter lesions, obesity, fatty liver and other diseases closely related to blood lipids were excluded. The study was approved by the ethics committee of West China Hospital of Sichuan University. All AD patients and control participants gave their written informed consent to participate in the investigation.

### Measurements

All blood samples were routinely collected in the early morning when patients were fasting. The serum lipid profiles, including TC, triglycerides (TG), LDL, and HDL, were measured by homogeneous enzyme colorimetry on Roche/Hitachi Cobas C analyzer. DNA was isolated from blood cells.

Samples were amplified by the polymerase chain reaction amplified samples (ABI 7500 FAST, Applied Biosystems, Thermo Fisher Scientific, Waltham, MA, United States). *APOE* haplotypes were determined according to the manufacturer’s instruction using an *APOE* haplotype determinating kit (Memorigen Biotech, Xiamen, China). The kit is based on fluorescent PCR technology, using three pairs of detection reagents that can specifically recognize two single nucleotide polymorphisms (RS429358 and RS7412) of *APOE* gene type 2, 3 and 4, respectively, to identify and amplify the samples. When the sample contained *APOE* allele matched with the amplification system, PCR amplification reaction would take place in the system. The exonuclease activity at the 5–3 end of DNA polymerase would degrade the fluorescent-labeled DNA molecular probe by enzyme digestion. After degradation by enzyme digestion, the probe could be stimulated with a fluorescence signal and detected by the monitoring system.

### Statistical Analyses

SPSS software 26.0 version (IBM, Armonk, NY, United States) was used for data analysis. The χ^2^ test was used to compare allele frequencies among groups. An independent *t*-test was used to compare lipid profiles in patients with different *APOE* haplotypes or sex. For those groups with significant age differences, age was adjusted by covariance analysis, for non-normal distribution data were used for non-parametric ANOVA (Kruskal–Wallis) and a non-parametric Mann–Whitney *U* test. Logistic regression was used to analyze the influences of various variables on the risk of disease. Two-tailed *p* < 0.05 was considered statistically significant. Table data are expressed as the means ± standard deviation (SD), and image values are expressed as the mean (standard error).

## Results

A total of 549 participants (298 AD patients, 251 HCs) were included in the study. The mean (SD) age of AD patients was 76.07 (7.12) years old, and the HCs were 65.85 (11.33) years old, so the age factor was adjusted in the subsequent lipid analyses (Supplementary Material 1 – adjusting for age). There were 113 male (185 female) people in AD and 103 male (148 female) people in HCs. In AD patients, the mean (SD) course of the disease was 2.5 (2.52) years, the mean (SD) of MMSE score was 18 (5.21), the mean (SD) of MOCA was 12.48 (3.56) (Table 1). There was no significant difference in statin use between the AD and the HCs groups (Table 1). Additional adjustments for the statin use did not change the statistical significance of the results of lipid analyses (Supplementary Material 2 – adjusting for age and the stain use).

**TABLE 1:** *APOE* gene and sex distribution in AD group and HCs group (mean ± standard deviation).

		AD (*n* = 298)	HCs (*n* = 251)	t/χ^2^	*p*-value
Age at examination, year		76.07 ± 7.12	65.85 ± 11.33	12.38	<0.001
Course of the disease, year		2.54 ± 2.52			
Education (>12 yearsr/<12 year)		134/164	131/120	2.85	0.09
MoCA scores		12.48 ± 5.21	24.00 ± 1.48	21.43	<0.001
MMSE scores		18 ± 3.56			
*APOEε4*	*APOEε4* +, n (%)	149 (50.00%)	38 (20.30%)	73.73	<0.001
*APOEε2*	*APOEε2* +, n (%)	18 (6.00%)	53 (21.10%)	27.50	<0.001
*APOEε3*	*APOEε3* +, n (%)	280 (93.96%)	236 (94.02%)	0.00	0.975
Sex	Male, n (%)	113 (37.92%)	103 (41.04%)	0.55	0.457
	Female, n (%)	185 (62.08%)	148 (58.96%)	0.55	0.457
Subtypes				79.340	<0.001
	ε2/ε2, ε2/ε3, ε3/ε3, n (%)	149 (41.20%)	213 (58.80%)	73.72	<0.001
	ε2/ε4, ε3/ε4, n (%)	131 (44.00%)	35 (13.90%)	58.19	<0.001
	ε4/ε4, n (%)	18 (6.00%)	3 (1.20%)	8.69	0.003
*APOE* haplotypes				99.46	<0.001
	ε2/ε2, n (%)	0	5 (0.90%)	5.99	0.02
	ε2/ε3, n (%)	18 (6.00%)	41 (16.30%)	15.05	<0.001
	ε3/ε3, n (%)	131 (44.00%)	167 (66.50%)	27.98	<0.001
	ε2/ε4, n (%)	0	7 (2.80%)	8.46	0.004
	ε3/ε4, n (%)	131 (44.00%)	28 (11.20%)	71.26	<0.001
	ε4/ε4, n (%)	18 (6.00%)	3 (1.20%)	8.69	0.003
The stain use		12 (4.00%)	8 (3.20%)	0.274	0.601
	*APOEε4* +	7 (4.70%)	2 (5.30%)	0.021	0.884
	*APOEε4* -	5 (3.40%)	6 (2.80%)	0.086	0.769
	*APOEε2* +	1 (5.60%)	1 (1.90%)	0.661	0.416
	*APOEε2* -	11 (3.90%)	7 (3.50%)	0.049	0.824
	Male	5 (4.40%)	3 (2.90%)	0.345	0.557
	Female	7 (3.80%)	5 (3.40%)	0.039	0.844

*AD, Alzheimer’s disease; HCs, healthy controls; APOEε4+, APOEε4 carriers; APOEε2+, APOEε2 carriers; APOEε3+, APOEε3 carriers; MMSE, the Mini-mental State Examination; MoCA, the Montreal Cognitive Assessment.*

*APOEε2 carriers included APOEε2/2, APOEε2/3, and APOEε2/4, and APOEε2 non-carriers included APOEε3/3, APOEε3/4, and APOEε4/4.*

*APOEε3 carriers included APOEε2/3, APOEε3/3, and APOEε3/4, and APOEε3 non-carriers included APOEε2/2, APOEε2/4, and APOEε4/4.*

*APOEε4 carriers included APOEε2/4, APOEε3/4, and APOEε4/4, and APOEε4 non-carriers included APOEε2/2, APOEε2/3, and APOEε3/3.*

### *APOE* Gene Analysis in Alzheimer’s Disease Patients and Healthy Controls

There were significant differences in the haplotype frequency of *APOE*ε*4*, *APOE*ε*2*, and *APOE*ε*3* between the AD and HCs (Table 1). A significantly higher proportion of *APOE*ε*4* carriers and a lower proportion of *APOE*ε*2* carriers were found in the AD group than in the HCs (Table 1). There was no significant difference in the proportion of *APOE*ε*3* carriers between AD group and HCs group, and the state of *APOE*ε*3* allele carrying had no effect on lipid profiles (Supplementary Material 3).

### Comparison of Lipid Profiles Between Alzheimer’s Disease Patients and Healthy Controls

**FIGURE 1:**
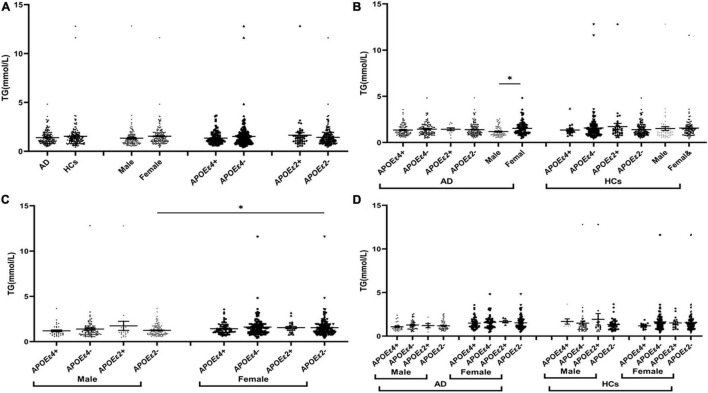
The levels of TG in different populations. *P < 0.05; Values are expressed as mean (standard error). AD, Alzheimer’s disease; HCs, healthy controls; *APOE*ε*4*+, *APOE*ε*4* carriers; *APOE*ε*2*+, *APOE*ε*2* carriers.

**FIGURE 2:**
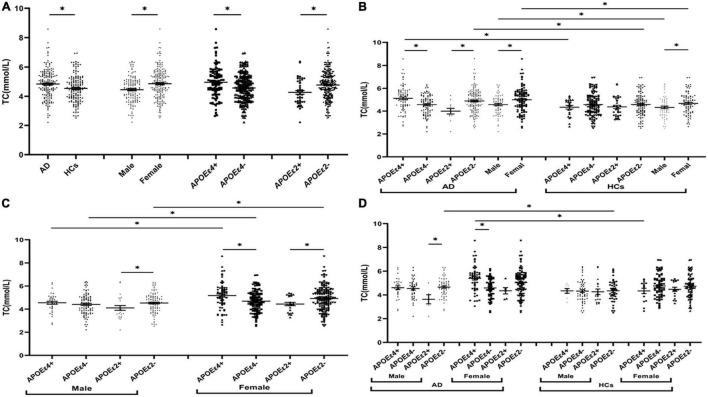
The levels of TC in different populations. *P < 0.05; Values are expressed as mean (standard error). AD, Alzheimer’s disease; HCs, healthy controls; *APOE*ε*4*+, *APOE*ε*4* carriers; *APOE*ε*2*+, *APOE*ε*2* carriers.

**FIGURE 3:**
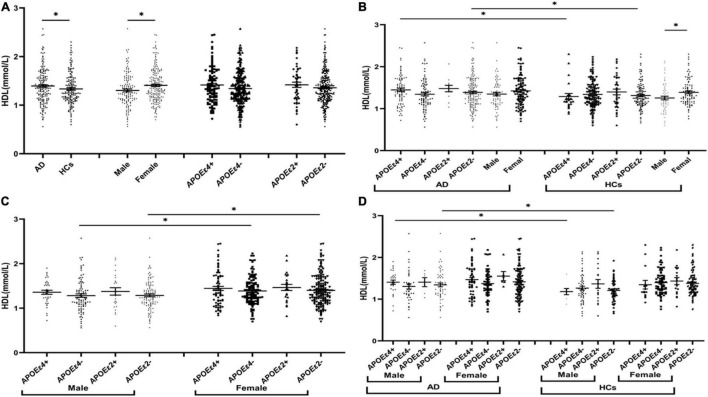
The levels of LDL in different populations. *P < 0.05; Values are expressed as mean (standard error). AD, Alzheimer’s disease; HCs, healthy controls; *APOE*ε*4*+, *APOE*ε*4* carriers; *APOE*ε*2*+, *APOE*ε*2* carriers.

**FIGURE 4:**
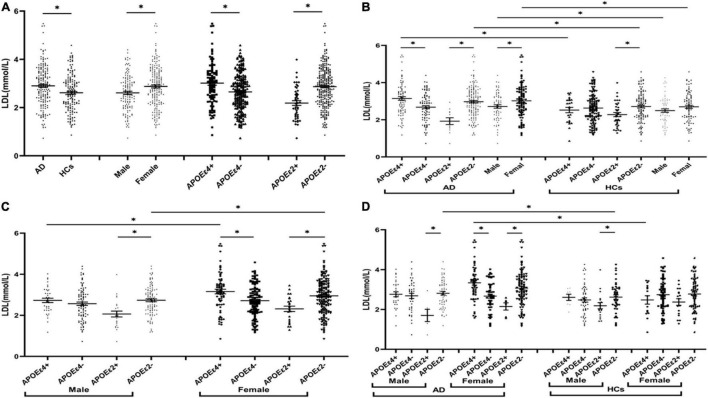
The levels of HDL in different populations. *P < 0.05; Values are expressed as mean (standard error). AD, Alzheimer’s disease; HCs, healthy controls; *APOE*ε*4*+, *APOE*ε*4* carriers; *APOE*ε*2*+, *APOE*ε*2* carriers.

The levels of TG were insignificant between AD and HCs groups (Figure 1). The levels of TC and LDL in the AD group were higher than those in the HCs group (Figures 2A, 3A). In the subgroup analysis based on *APOE* alleles, AD patients carrying *APOE*ε*4* had higher levels of TC and LDL than HCs with *APOE*ε*4* allele; AD patients without *APOE*ε*2* allele had increased TC and LDL levels than HCs without *APOE*ε*2* allele (Figures 2B, 3B). In subgroup analysis based on sex, TC and LDL levels in the AD group were significantly higher than in the HCs group in both sexes (Figures 2B, 3B). A subgroup analysis combined sex and *APOE* haplotypes showed that TC and LDL levels were higher in AD patients than in HCs, which were only found in female *APOE*ε*4* carriers or male *APOE*ε*2* non-carriers (Figures 2D, 3D). In addition, serum HDL level in the AD group was higher than those in the HCs group (Figure 4A). Subgroup analysis showed that the change of HDL level was found in populations with *APOE*ε*4* allele and without *APOE*ε*2* allele (Figure 4B), as well as in male non-*APOE*ε*2* carriers and male *APOE*ε*4* carriers (Figure 4D).

### Comparison of Lipid Profiles Between *APOE*ε*4* Allele-Carriers and Non-*APOE*ε*4* Allele-Carriers

The levels of TC and LDL in the *APOE*ε*4* carriers were higher than those in non-*APOE*ε*4* carriers (Figures 2A, 3A). In the subgroup analysis based on disease, we found that AD patients with *APOE*ε*4* had significantly higher levels of TC and LDL than AD patients without *APOE*ε*4* (Figures 2B, 3B). No differences in the levels of TC and LDL between HCs with and without *APOE*ε*4* (Figures 2B, 3B). Furthermore, In the subgroup analysis based on sex, significantly higher levels of TC and LDL were only found in female AD patients with *APOE*ε*4* than without *APOE*ε*4* female AD patients (Figures 2D, 3D). However, no differences were found between male AD patients with and without *APOE*ε*4* (Figures 2D,  3D).

### Comparison of Lipid Profiles Between *APOE*ε*2* Allele-Carriers and Non-*APOE*ε*2* Allele-Carriers

The levels of TC and LDL in the *APOE*ε*2* carriers were lower than those in non-*APOE*ε*2* carriers (Figures 2A, 3A). In the subgroup analysis based on sex and disease, similar changes in LDL levels were found in almost all subgroups except the female control population (Figures 3B–D). The TC levels were lower in AD patients with the *APOE*ε*2* allele than in AD patients without the *APOE*ε*2* allele, but no such change was observed in the HCs (Figure 2B). In addition, the TC of *APOE*ε*2* carriers was lower than that of non-carriers in the male AD population, but there was no such change in the female AD population, female HCs, and male HCs (Figure 2D).

### Comparison of Lipid Profiles Between the Male and the Female

The TC, HDL, and LDL levels in the female population were higher than the male population (Figures 2A, 3A, 4A). In order to exclude the effect of disease and *APOE* gene, subgroup analyses were performed, and the results showed that sex had similar effects on TC and LDL in AD patients, *APOE*ε*4* carriers, and *APOE*ε*2* non-carriers (Figures 2B,C, 3B,C). In the control participants and *APOE*ε*4* non-carriers, TC and HDL in the female population were higher than the male population (Figures 2B,C, 4B,C).

### Complex Interactions Exist Between Alzheimer’s Disease, *APOE* Haplotypes, Lipid Profiles, and Sex

Age, gender, disease course of AD, *APOE*ε*4*, and blood lipids level were considered influencing factors for Logistics regression analysis, and we found that age and *APOE4* were important risk factors, while the blood lipids were not significantly related with AD. When TG, TC, HDL, and LDL were taken as targets to study the relationship with AD, no correlation was found between lipid profiles and AD through logistics regression analysis. However, in the subgroup analysis, adjusted *APOE* and sex, we found that LDL increased the risk of AD in females with the *APOE*ε*4* allele, that is, for every 1 unit increase in LDL, the risk of AD increased 898.46 times in the female population with *APOE*ε*4* (*P* = 0.04). Therefore, the increased LDL level may increase the risk of AD in female people carrying *APOE*ε*4*.

## Discussion

This study found that LDL and TC serum levels in AD patients were higher than those in HCs, consistent with previous studies (Wang et al., 2020). In addition, we found higher HDL levels in AD patients compared to controls. A prospective study published in 2021 reported very high serum HDL cholesterol levels as an independent risk factor for either dementia or AD and suggested that elevated HDL may serve as a serum biomarker for assessing the risk of dementia (Kjeldsen et al., 2021). However, a prospective study with approximately 7,000 French people found that HDL was not associated with AD (Schilling et al., 2017). A meta-analysis that combined all relevant studies before 2017 showed that HDL was not associated with AD in later life (Anstey et al., 2017). Marsillach et al. (2020) point out that functional HDL is more important in disease rather than HDL cholesterol levels. Therefore, more attention should be paid to the interaction between HDL functional subtypes and AD.

In the AD population, the TC and LDL levels were increased in the *APOE*ε*4* carriers compared to the non-*APOE*ε*4* carriers, and this alteration was not found in the control population. In *APOE*ε*4* allele carriers, the TC and LDL levels in the AD patients were higher than those in control participants, but these differences in TC and LDL were not found in non-*APOE*ε*4* carriers. These findings indicated that the involvement of *APOE*ε*4* in AD could be associated with lipid profiles. Similarly, Wang et al. (2020) found that the levels of TC in the AD population carrying *APOE*ε*4* were higher than those without *APOE*ε*4*, while no such change was observed in healthy controls. However, Raygani et al. (2006) found these lipid changes between *APOE*ε*4* carriers and non-*APOE*ε*4* carriers in both AD and controls. In addition, we also found that LDL may increase the risk of AD in females with the *APOE*ε*4* allele. A previous study showed that the association of elevated midlife TC level with late-life AD was not altered after adjusting for the *APOE*ε*4* allele (Kivipelto et al., 2002), but another study showed decreasing TC after midlife may represent a risk marker for late-life cognitive impairment (Solomon et al., 2007), and these studies did not take into account the role of sex. Therefore, further research and exploration are needed to verify if the effect of *APOE*ε*4* on lipid profiles is gender-specific in AD.

In subgroup analyses based on sex showed that TC and LDL in the AD group were higher than those of the control group in both male and female populations. In the female population, TC and LDL were higher in *APOE*ε*4* carriers than in non-*APOE*ε*4* carriers, but no change was found in the male population. Several recent studies have shown that sex can alter the risk of the *APOE*ε*4* allele, and female people with *APOE*ε*4* have a higher risk of AD than male carriers (Kim et al., 2015). Women aged 65–75 with *APOE*ε*4* had a higher risk of AD than men and had higher levels of tau in the cerebrospinal fluid (CSF) (Liu et al., 2021). A significant sex-specific association was found between CSF apolipoprotein E and AD biomarkers in Liu’s study. In women, baseline CSF apolipoprotein E was significantly associated with longitudinal changes in baseline CSF Aβ and tau, but no longitudinal association was observed in men (Liu et al., 2021). In addition, a recent study on brain metabolism found that the exponential function of brain metabolism in females declines more rapidly than the linear function in males when young and more slowly in old age. The explanation for this could be a greater brain reserve in men, indicating that men have a greater capacity to withstand more pathology than women (Zhang et al., 2017). Our study found that the effect of the *APOE*ε*4* allele on TC and LDL metabolism is significantly altered in female AD patients, but not in male AD patients, so we think the difference may also be related to metabolic reserve.

The effect of the *APOE*ε*4* allele on lipid profiles in women was greater than that in men, but the effect of the *APOE*ε*2* allele on lipid profiles was different from the *APOE*ε*4* allele. In this study, the individuals were stratified according to the presence of the *APOE*ε*2* allele, and we found that the role of the *APOE*ε*2* allele in lipid levels was affected by the disease of AD. In the AD population, the TC level in *APOE*ε*2* carriers was lower than those in non-carriers, but these changes were not found in the HCs. Although the TC level of *APOE*ε*2* carriers was lower than that of non-carriers in both male and female populations, such change was only found in the male AD population, but not in the male HCs, female HCs, and female AD population. Therefore, we hypothesized that reducing TC by *APOE*ε*2* allele seems to be more biased in male AD populations. Human studies have shown that the *APOE*ε*2* allele is associated with decreased Aβ deposition in the brain of non-dementia elderly individuals and AD patients, and protects against cognitive impairment in individuals over 90 years of age with high levels of Aβ in the brain (Serrano-Pozo et al., 2015). *In vitro* and *in vivo* studies have shown that *APOE*ε*2* provides protection independent of Aβ pathology through multiple potential pathways, including the regulatory role of *APOE*ε*2* in lipid metabolism (Huang et al., 2019).

It is well known that the main component of senile plaques is Aβ, which is the main pathological basis of AD. Aβ is produced by the double cleavage of amyloid precursor protein (APP) by β-secretase and γ-secretase. APP, β-secretase and γ-secretase are located in lipid rafts, a type of membrane-bound cholesterol, where APP metabolism occurs. The level of 27-OH cholesterol circulating in the blood is proportional to the level of cholesterol, and there is a concentration-driven flux of 27-OH cholesterol circulating into the brain. The accumulation of 27-OH cholesterol was found to be the most significant change in the brain cholesterol profile of AD patients, and part of this accumulation may be influenced by circulating cholesterol levels. High levels of 27-OH cholesterol increase the formation of Aβ by antagonizing the inhibition of 24S-hydroxyl cholesterol (Feringa and van der Kant, 2021). It is speculated that the increase of tag-rich lipoprotein in the ε4 vector *APOE* may increase its affinity for LDLR, which ultimately reduces LDL intake and increases circulating plasma cholesterol. *APOE*ε*4* has been shown to contribute to AD susceptibility by disrupting lipid and cholesterol levels. One study used correlation analysis to detect gene expression patterns in *APOE*ε*4*-positive and *APOE*ε*4*-negative elderly men and women. A large number of genes with the same *APOE* expression pattern were found in *APOE*ε*4*-positive individuals but not in *APOE*ε*4*-negative individuals. *APOE*ε*4*-positive women tended to be larger than *APOE*ε*4*-positive men and *APOE*ε*4*-negative controls. The classification of genes is concerned with hormones involved in oxidation, inflammatory lipid metabolism, and immune processes. A significant triple interaction was observed in the brain region/sex/genotype of γ-secretase, which is closely associated with AD and is thought to play a role in APP processing (Hsu et al., 2019). This may explain the finding in this study that high LDL levels may increase the risk of AD in female people with the *APOE*ε*4* allele. Therefore, more attention should be paid to lipid profiles of different genders and APOE genotypes in the future study of AD, especially women who carry the *APOE4* allele.

In general, cholesterol and *APOE*ε*4* gene are major factors affecting the development of AD, but current studies have found that statins may have a protective effect before the onset of AD, and once the onset of the disease, statins become inefficient or ineffective (Li et al., 2010). A longitudinal study in 2021 evaluated the association between statin use (the time-varying status and the dose-response relationship) and the incidence of AD, and found that statin use was not associated with an increased incidence of AD (Jeong et al., 2021). In this study, The proportion of statin use was small and adjustment for statin use did not significantly affect lipid profile results. However, this study was a cross-sectional result, and it is worthwhile to further explore whether statins benefit female AD patients with *APOE4* allele in longitudinal follow-up because these subpopulations have more significant changes in TC and LDL.

## Conclusion

The TC and LDL levels of *APOE*ε*4* allele carriers were higher than those of non-carriers, and the effect was more significant in the AD and female population. High LDL levels may increase the risk of AD in female people with the *APOE*ε*4* allele. The TC levels in *APOE*ε*2* allele carriers were lower than those in non-carriers, and the effect was more significant in the male AD population. Further prospective studies focusing on the relationship between the *APOE* gene, sex, lipid profiles, and AD are essential to confirm our findings, and special attention should be paid to female AD patients with the *APOE*ε*4* allele and male AD patients carrying the *APOE*ε*2* allele when regulating the blood lipids.

## Limitations

This study has the following limitations. First, due to the large difference in whether AD patients take medication, type of medication, and dose, there is no stratification according to medication. Second, this study did not analyze the relationship between lipid profiles and AD severity due to limited data. Thirdly, This study was cross-sectional and lacks the dynamic changes and correlation of lipid profiles with time and disease. Fourthly, this study is a single-center, small-sample study, which needs to be verified by a larger sample and multi-center study.

## Data Availability Statement

The original contributions presented in the study are included in the article/Supplementary Material, further inquiries can be directed to the corresponding author/s.

## Ethics Statement

The studies involving human participants were reviewed and approved by the Ethics Committee of West China Hospital of Sichuan University. The patients/participants provided their written informed consent to participate in this study.

## Author Contributions

JF contributed to the compilation of articles and data analysis. YH and TB contributed to the selection and data entry of healthy controls. RO, QW, YC, and JY contributed to the screening and data entry of AD patients. XC and HS contributed to the review, editing and scientific research thinking, and methods. All authors read and approved the manuscript.

## Conflict of Interest

The authors declare that the research was conducted in the absence of any commercial or financial relationships that could be construed as a potential conflict of interest.

## Publisher’s Note

All claims expressed in this article are solely those of the authors and do not necessarily represent those of their affiliated organizations, or those of the publisher, the editors and the reviewers. Any product that may be evaluated in this article, or claim that may be made by its manufacturer, is not guaranteed or endorsed by the publisher.

## Supplementary Material

**Supplementary Material 1**

Supplementary Table 1 Comparison of lipids between AD and HCs groups (Adjusting for age); Values are expressed as mean (standard deviation); AD, Alzheimer’s disease; HCs, healthy controls; *APOE*ε*4*+, *APOE*ε*4* carriers; *APOE*ε*2*+, *APOE*ε*2* carriers; Male and *APOE*ε*4*+, male *APOE*ε*4* carriers; Male and *APOE*ε*4−*, male *APOE*ε*4* non-carriers*;* Female and *APOE*ε*4*+, female *APOE*ε*4* carrier*;* Female and *APOE*ε*4−*, female *APOE*ε*4* non-carrier*;* Male and *APOE*ε*2*+, male *APOE*ε*2* carriers; Male and *APOE*ε*2−*, male *APOE*ε*2* non-carriers*;* Female and *APOE*ε*2*+, female *APOE*ε*2* carrier*;* Female and *APOE*ε*2−*, female *APOE*ε*2* non-carrier.

Supplementary Table 2 Comparison of lipids between *APOE*ε*4*+ group and *APOE*ε*4−* group (Adjusting for age); Values are expressed as mean (standard deviation); AD, Alzheimer’s disease; HCs, healthy controls; Male and AD, male patients with Alzheimer’s disease; Female and AD, female patients with Alzheimer’s disease; Male and HCs, male healthy controls; Female and HCs, healthy female controls.

Supplementary Table 3 Comparison of lipids between *APOE*ε*2*+ group and *APOE*ε*2−* group (Adjusting for age); Values are expressed as mean (standard deviation); AD, Alzheimer’s disease; HCs, healthy controls; Male and AD, male patients with Alzheimer’s disease; Female and AD, female patients with Alzheimer’s disease; Male and HCs, male healthy controls; Female and HCs, healthy female controls.

Supplementary Table 4 Comparison of lipids between male and female (Adjusting for age); Values are expressed as mean (standard deviation); AD, Alzheimer’s disease; HCs, healthy controls; *APOE*ε*4*+, *APOE*ε*4* carrier.

**Supplementary Material 2**

Supplementary Table 1 Comparison of lipids between AD and HCs groups (Adjusting for age and the stain use); Values are expressed as mean (standard deviation); AD, Alzheimer’s disease; HCs, healthy controls; *APOE*ε*4*+, *APOE*ε*4* carriers; *APOE*ε*2*+, *APOE*ε*2* carriers; Male and *APOE*ε*4*+, male *APOE*ε*4* carriers; Male and *APOE*ε*4−*, male *APOE*ε*4* non-carriers*;* Female and *APOE*ε*4*+, female *APOE*ε*4* carrier*;* Female and *APOE*ε*4−*, female *APOE*ε*4* non-carrier*;* Male and *APOE*ε*2*+, male *APOE*ε*2* carriers; Male and *APOE*ε*2−*, male *APOE*ε*2* non-carriers*;* Female and *APOE*ε*2*+, female *APOE*ε*2* carrier*;* Female and *APOE*ε*2−*, female *APOE*ε*2* non-carrier.

Supplementary Table 2 Comparison of lipids between *APOE*ε*4*+ group and *APOE*ε*4−* group (Adjusting for age and the stain use); Values are expressed as mean (standard deviation); AD, Alzheimer’s disease; HCs, healthy controls; Male and AD, male patients with Alzheimer’s disease; Female and AD, female patients with Alzheimer’s disease; Male and HCs, male healthy controls; Female and HCs, healthy female controls.

Supplementary Table 3 Comparison of lipids between *APOE*ε*2*+ group and *APOE*ε*2−* group (Adjusting for age and the stain use); Values are expressed as mean (standard deviation); AD, Alzheimer’s disease; HCs, healthy controls; Male and AD, male patients with Alzheimer’s disease; Female and AD, female patients with Alzheimer’s disease; Male and HCs, male healthy controls; Female and HCs, healthy female controls.

Supplementary Table 4 Comparison of lipids between male and female (Adjusting for age and the stain use); Values are expressed as mean (standard deviation); AD, Alzheimer’s disease; HCs, healthy controls; *APOE*ε*4*+, *APOE*ε*4* carrier.

**Supplementary Material 3**

Supplementary Table 1 Comparison of lipids between AD and HCs groups (Adjusting for age); Values are expressed as mean (standard deviation); AD, Alzheimer’s disease; HCs, healthy controls; *APOE*ε*3*+, *APOE*ε*3* carriers; Male and *APOE*ε*3*+, male *APOE*ε*3* carriers; Male and *APOE*ε*3−*, male *APOE*ε*3* non-carriers*;* Female and *APOE*ε*3*+, female *APOE*ε*3* carrier*;* Female and *APOE*ε*3−*, female *APOE*ε*3* non-carrier.

Supplementary Table 2 Comparison of lipids between *APOE*ε*3*+ group and *APOE*ε*3−* group (Adjusting for age); Values are expressed as mean (standard deviation); AD, Alzheimer’s disease; HCs, healthy controls.
