# Supplementary material for: Effects of Sex on the Relationship Between Apolipoprotein E Gene and Serum Lipid Profiles in Alzheimer’s Disease
Source: Front Aging Neurosci. 2022 May 30;14:844066. doi: 10.3389/fnagi.2022.844066 (PMC9190463; doi:10.3389/fnagi.2022.844066)
Supplement: Supplementary Table 1 — Comparison of lipids between AD and HCs groups (Adjusting for age); Values are expressed as mean (standard deviation); AD, Alzheimer’s disease; HCs, healthy controls; APOEε4+, APOEε4 carriers; APOEε2+, APOEε2 carriers; Male and APOEε4+, male APOEε4 carriers; Male and APOEε4−, male APOEε4 non-carriers; Female and APOEε4+, female APOEε4 carrier; Female and APOEε4−, female APOEε4 non-carrier; Male and APOEε2+, male APOEε2 carriers; Male and APOEε2−, male APOEε2 non-carriers; Female and APOEε2+, female APOEε2 carrier; Female and APOEε2−, female APOEε2 non-carrier. [file Table_1.docx]

| Table S1: Comparison of lipids between AD and HCs groups (Adjusting for age) (mean±sd) | | | | | | | | | | | | | | | | | | | | | | | | |
| --- | --- | --- | --- | --- | --- | --- | --- | --- | --- | --- | --- | --- | --- | --- | --- | --- | --- | --- | --- | --- | --- | --- | --- | --- |
|  | n | | TG (mmol/L) | | | | TC (mmol/L) | | | | HDL (mmol/L) | | | | | LDL (mmol/L) | | | | LDL/HDL | | | |  |
|  | HCs | AD | HCs | AD | P | Logistics (Exp, P) | HCs | AD | P | Logistics (Exp, P) | | HCs | AD | P | Logistics (Exp, P) | HCs | AD | P | Logistics (Exp, P) | HCs | AD | P | Logistics (Exp, P) | predictions correct (%) |
| overall | 251 | 298 | 1.54±1.39 | 1.40±0.67 | 0.73 | 0.91(0.66) | 4.54±0.93 | 4.84±1.01 | 0.00 | 0.90(0.86) | | 1.33±0.33 | 1.39±0.36 | 0.03 | 3.15(0.27) | 2.62±0.77 | 2.90±0.85 | 0.00 | 1.14(0.85) | 2.06±0.71 | 2.21±0.86 | 0.15 | 1.70(0.33) | 59.00 |
| *APOEε4+* | 38 | 149 | 1.35±0.60 | 1.35±0.63 | 0.79 | 1.13(0.85) | 4.34±0.77 | 5.13±1.08 | 0.00 | 0.57(0.77) | | 1.29±0.35 | 1.45±0.35 | 0.02 | 0.85(0.95) | 2.52±0.70 | 3.15±0.88 | 0.00 | 12.01(0.27) | 2.07±0.70 | 2.28±0.84 | 0.36 | 0.29(0.35) | 78.00 |
| *APOEε4-* | 213 | 149 | 1.57±1.49 | 1.44±0.71 | 0.97 | 0.93(0.77) | 4.57±0.96 | 4.57±0.85 | 0.59 | 0.79(0.67) | | 1.34±0.33 | 1.34±0.36 | 0.69 | 5.35(0.15) | 2.63±0.78 | 2.68±0.77 | 0.61 | 0.58(0.45) | 2.05±0.72 | 2.14±0.88 | 0.58 | 3.08(0.07) | 61.80 |
| *APOEε2+* | 53 | 18 | 1.73±2.03 | 1.44±0.52 | 0.95 | 0.88(0.88) | 4.37±0.86 | 4.00±0.86 | 0.31 | 7.09(0.47) | | 1.40±0.40 | 1.48±0.27 | 0.50 | 0.00(0.04) | 2.28±0.66 | 1.93±0.64 | 0.09 | 367.75(0.18) | 1.74±0.61 | 1.29±0.37 | 0.02 | 0.00(0.03) | 76.60 |
| *APOEε2-* | 198 | 280 | 1.48±1.14 | 1.40±0.68 | 0.73 | 0.88(0.59) | 4.59±0.59 | 4.90±0.99 | 0.00 | 1.07(0.91) | | 1.31±0.30 | 1.39±0.36 | 0.02 | 5.49(0.15) | 2.72±0.77 | 2.97±0.83 | 0.01 | 0.71(0.63) | 2.15±0.71 | 2.27±0.85 | 0.22 | 2.36(0.16) | 61.30 |
| male | 103 | 113 | 1.51±1.59 | 1.19±0.52 | 0.33 | 0.33(0.06) | 4.32±0.90 | 4.57±0.84 | 0.02 | 7.47(0.15) | | 1.25±0.34 | 1.35±0.35 | 0.10 | 0.79(0.90) | 2.50±0.73 | 2.72±0.76 | 0.02 | 0.10(0.14) | 2.10±0.74 | 2.17±0.89 | 0.39 | 2.25(0.30) | 67.20 |
| female | 148 | 185 | 1.56±1.25 | 1.53±0.72 | 0.69 | 1.20(0.58) | 4.69±0.93 | 5.01±1.07 | 0.04 | 0.50(0.44) | | 1.39±0.31 | 1.42±0.36 | 0.12 | 4.80(0.31) | 2.70±0.79 | 3.02±0.89 | 0.05 | 2.09(0.50) | 2.03±0.69 | 2.23±0.85 | 0.42 | 1.72(0.51) | 60.60 |
| Male & *APOEε4+* | 13 | 58 | 1.68±0.85 | 1.07±0.47 | 0.05 | 0.10(0.09) | 4.34±0.57 | 4.61±0.84 | 0.05 | 33.88(0.38) | | 1.18±0.21 | 1.41±0.28 | 0.03 | 489.40(0.51) | 2.61±0.44 | 2.76±0.68 | 0.06 | 0.00(0.20) | 2.25±0.44 | 2.03±0.67 | 0.90 | 39.84(0.37) | 81.10 |
| Male & *APOEε4-* | 90 | 55 | 1.48±1.68 | 1.27±0.55 | 0.72 | 0.30(0.09) | 4.32±0.94 | 4.54±0.85 | 0.10 | 16.78(0.11) | | 1.26±0.35 | 1.31±0.39 | 0.54 | 0.51(0.75) | 2.48±0.76 | 2.68±0.82 | 0.12 | 0.03(0.07) | 2.08±0.78 | 2.26±1.02 | 0.25 | 3.59(0.13) | 61.70 |
| Female & *APOEε4+* | 25 | 91 | 1.18±0.32 | 1.50±0.65 | 0.18 | 11.10(0.60) | 4.34±0.88 | 5.39±0.10 | 0.00 | 0.02(0.16) | | 1.35±0.40 | 1.47±0.39 | 0.12 | 9.66(0.54) | 2.48±0.81 | 3.34±0.90 | 0.00 | 898.46(0.04) | 1.97±0.81 | 2.41±0.90 | 0.18 | 0.13(0.27) | 80.60 |
| Female & *APOEε4-* | 123 | 94 | 1.64±1.35 | 1.57±0.79 | 0.64 | 1.11(0.75) | 4.76±0.93 | 4.59±0.86 | 0.38 | 0.50(0.35) | | 1.40±0.30 | 1.37±0.33 | 0.94 | 5.82(0.33) | 2.74±0.79 | 2.67±0.74 | 0.32 | 0.77(0.81) | 2.04±0.67 | 2.04±0.75 | 0.51 | 3.00(0.32) | 60.30 |
| Male & *APOEε2+* | 25 | 8 | 1.93±2.78 | 1.20±0.60 | 0.80 | 0.06(0.28) | 4.27±0.97 | 3.64±0.94 | 0.20 | 11669.66(0.22) | | 1.37±0.45 | 1.41±0.26 | 0.95 | 0.00(0.09) | 2.19±0.65 | 1.70±0.76 | 0.10 | 0.48(0.94) | 1.73±0.57 | 1.19±0.44 | 0.07 | 0.00(0.12) | 87.50 |
| Male & *APOEε2-* | 78 | 105 | 1.34±0.66 | 1.19±0.52 | 0.19 | 0.36(0.12) | 4.35±0.88 | 4.66±0.78 | 0.00 | 6.52(0.24) | | 1.21±0.27 | 1.34±0.36 | 0.02 | 16.93(0.30) | 2.62±0.73 | 2.81±0.69 | 0.01 | 0.04(0.09) | 2.25±0.75 | 2.26±0.87 | 0.54 | 7.01(0.08) | 66.40 |
| Female & *APOEε2+* | 28 | 10 | 1.51±0.69 | 1.68±0.31 | 0.67 | 4.02(0.32) | 4.48±0.72 | 4.35±0.65 | 0.89 | 3.57(0.73) | | 1.43±0.35 | 1.55±0.27 | 0.15 | 0.00(0.23) | 2.37±0.68 | 2.15±0.44 | 0.55 | 3354.72(0.14) | 1.75±0.66 | 1.40±0.28 | 0.11 | 0.00(0.14) | 73.90 |
| Female & *APOEε2-* | 120 | 175 | 1.57±1.35 | 1.52±0.74 | 0.67 | 1.02(0.96) | 4.74±0.97 | 5.05±1.08 | 0.10 | 0.78(0.73) | | 1.38±0.31 | 1.42±0.37 | 0.24 | 3.99(0.37) | 2.78±0.80 | 3.07±0.89 | 0.11 | 1.12(0.91) | 2.09±0.69 | 2.28±0.84 | 0.46 | 2.08(0.41) | 61.10 |

| Table S2: Comparison of lipids between *APOEε4+* group and *APOEε4-* group (Adjusting for age) (mean±sd) | | | | | | | | | | | | | | | | | | | | | | | |
| --- | --- | --- | --- | --- | --- | --- | --- | --- | --- | --- | --- | --- | --- | --- | --- | --- | --- | --- | --- | --- | --- | --- | --- |
|  | n | | TG (mmol/L) | | | | TC (mmol/L) | | | | HDL (mmol/L) | | | | LDL (mmol/L) | | | | LDL/HDL | | | |  |
|  | *APOEε4+* | *APOEε4-* | *APOEε4+* | *APOEε4-* | P | logistics (Exp, P) | *APOEε4+* | *APOEε4-* | P | logistics (Exp, P) | *APOEε4+* | *APOEε4-* | P | logistics (Exp, P) | *APOEε4+* | *APOEε4-* | P | logistics (Exp, P) | *APOEε4+* | *APOEε4-* | P | logistics (Exp, P) | predictions correct (%) |
| overall | 187 | 362 | 1.35±0.62 | 1.52±1.23 | 0.31 | 1.42(0.21) | 4.96±1.07 | 4.57±0.91 | 0.00 | 0.79(0.66) | 1.41±0.36 | 1.34±0.34 | 0.06 | 1.77(0.61) | 3.01±0.88 | 2.65±0.78 | 0.00 | 0.51(0.33) | 2.24±0.82 | 2.09±0.79 | 0.15 | 1.55(0.46） | 66.30 |
| AD | 149 | 149 | 1.35±0.63 | 1.44±0.71 | 0.44 | 1.58(0.20) | 5.13±0.18 | 4.57±0.85 | 0.00 | 0.60(0.38) | 1.45±0.35 | 1.34±0.36 | 0.07 | 6.45(0.18) | 3.15±0.88 | 2.68±0.77 | 0.00 | 0.28(0.12) | 2.28±0.84 | 2.14±0.88 | 0.26 | 3.67(0.09) | 62.90 |
| HCs | 38 | 213 | 1.35±0.60 | 1.57±1.49 | 0.62 | 1.41(0.61) | 4.34±0.77 | 4.57±0.96 | 0.24 | 1.11(0.96) | 1.29±0.35 | 1.34±0.33 | 0.53 | 0.56(0.83) | 2.52±0.70 | 2.63±0.78 | 0.53 | 2.28(0.69) | 2.07±0.70 | 2.05±0.72 | 0.95 | 0.35(0.37) | 84.80 |
| Male | 71 | 145 | 1.20±0.61 | 1.39±1.32 | 0.51 | 0.49(0.22) | 4.55±0.79 | 4.42±0.91 | 0.33 | 12.31(0.12) | 1.36±0.28 | 1.28±0.37 | 0.26 | 1.13(0.96) | 2.73±0.63 | 2.57±0.79 | 0.22 | 0.01(0.02) | 2.08±0.63 | 2.16±0.89 | 0.70 | 7.78(0.09) | 71.00 |
| Female | 116 | 217 | 1.43±0.61 | 1.61±1.15 | 0.29 | 2.16(0.05) | 5.17±1.14 | 4.69±0.90 | 0.01 | 0.45(0.30) | 1.44±0.39 | 1.39±0.31 | 0.21 | 0.75(0.85) | 3.16±0.95 | 2.71±0.76 | 0.01 | 1.91(0.50) | 2.32±0.89 | 2.04±0.70 | 0.03 | 0.46(0.35) | 66.70 |
| Male & AD | 58 | 55 | 1.07±0.47 | 1.27±0.55 | 0.11 | 0.80(0.80) | 4.61±0.84 | 4.54±0.85 | 0.71 | 9.95(0.24) | 1.41±0.28 | 1.31±0.39 | 0.23 | 2.29(0.76) | 2.76±0.68 | 2.68±0.82 | 0.65 | 0.01(0.07) | 2.03±0.67 | 2.26±1.02 | 0.30 | 10.07(0.10) | 72.50 |
| Male & HCs | 13 | 90 | 1.68±0.85 | 1.48±1.68 | 0.82 | 0.09(0.04) | 4.34±0.57 | 4.32±0.94 | 0.96 | 1584.62(0.04) | 1.18±0.21 | 1.26±0.35 | 0.53 | 0.19(0.79) | 2.61±0.44 | 2.48±0.76 | 0.69 | 0.00(0.03) | 2.25±0.44 | 2.08±0.78 | 0.60 | 11.98(0.40) | 87.10 |
| Female & AD | 91 | 94 | 1.49±0.65 | 1.57±0.79 | 0.71 | 1.78(0.20) | 5.39±0.10 | 4.59±0.86 | 0.00 | 0.42(0.22) | 1.47±0.39 | 1.37±0.33 | 0.27 | 3.34(0.53) | 2.34±0.90 | 2.67±2.74 | 0.00 | 0.58(0.60) | 2.41±0.90 | 2.04±0.75 | 0.01 | 1.59(0.66) | 67.90 |
| Female & HCs | 25 | 123 | 1.18±0.32 | 1.64±1.35 | 0.23 | 14.14(0.02) | 4.33±0.88 | 4.76±0.93 | 0.10 | 0.07(0.29) | 1.35±0.40 | 1.40±0.30 | 0.76 | 0.21(0.69) | 2.48±0.81 | 2.74±0.79 | 0.12 | 1295.49(0.02) | 1.97±0.81 | 2.04±0.67 | 0.45 | 0.00(0.02) | 83.10 |

| Table S3: Comparison of lipids between *APOEε2+* group and *APOEε2-* group (Adjusting for age) (mean±sd) | | | | | | | | | | | | | | | | | | | | | | | |
| --- | --- | --- | --- | --- | --- | --- | --- | --- | --- | --- | --- | --- | --- | --- | --- | --- | --- | --- | --- | --- | --- | --- | --- |
|  | n | | TG (mmol/L) | | | | TC (mmol/L) | | | | HDL (mmol/L) | | | | LDL (mmol/L) | | | | LDL/HDL | | | |  |
|  | *APOEε2+* | *APOEε2-* | *APOEε2+* | *APOEε2-* | P | logistics (Exp, P) | *APOEε2+* | *APOEε2-* | P | logistics (Exp, P) | *APOEε2+* | *APOEε2-* | P | logistics (Exp, P) | *APOEε2+* | *APOEε2-* | P | logistics (Exp, P) | *APOEε2+* | *APOEε2-* | P | logistics (Exp, P) | predictions correct (%) |
| overall | 71 | 478 | 1.65±1.77 | 1.43±0.90 | 0.29 | 2.68(0.03) | 4.27±0.86 | 4.77±0.99 | 0.00 | 0.02(0.00) | 1.42±0.37 | 1.36±0.34 | 0.29 | 34.06(0.06) | 2.19±0.67 | 2.87±0.81 | 0.00 | 103.66(0.00) | 1.63±0.59 | 2.22±0.80 | 0.00 | 2.53(0.40) | 86.00 |
| AD | 18 | 280 | 1.44±0.52 | 1.40±0.68 | 0.85 | 1.14(0.90) | 3.99±0.86 | 4.90±0.99 | 0.00 | 0.02(0.21) | 1.48±0.27 | 1.39±0.36 | 0.38 | 4615.18(0.08) | 1.93±0.64 | 2.97±0.83 | 0.00 | 1.63(0.90) | 1.29±0.37 | 2.27±0.85 | 0.00 | 7395.39(0.04) | 94.90 |
| HCs | 53 | 198 | 1.73±2.03 | 1.48±1.14 | 0.43 | 4.39(0.02) | 4.37±0.86 | 4.95±0.95 | 0.20 | 0.01(0.01) | 1.40±0.40 | 1.31±0.30 | 0.19 | 79.96(0.10) | 2.28±0.66 | 2.72±0.77 | 0.00 | 339.43(0.00) | 1.74±0.61 | 2.15±0.71 | 0.00 | 1.75(0.68) | 79.50 |
| Male | 33 | 183 | 1.75±2.43 | 1.25±0.58 | 0.16 | 0.74(0.68) | 4.11±0.99 | 4.53±0.83 | 0.02 | 0.15(0.32) | 1.38±0.41 | 1.29±0.33 | 0.25 | 3.98(0.58) | 2.07±0.70 | 2.73±0.71 | 0.00 | 11.38(0.27) | 1.59±0.59 | 2.26±0.82 | 0.00 | 4.09(0.35) | 83.20 |
| Female | 38 | 295 | 1.55±0.61 | 1.54±1.03 | 0.91 | 7.31(0.00) | 4.44±0.69 | 4.92±1.04 | 0.03 | 0.00(0.00) | 1.46±0.33 | 1.40±0.34 | 0.49 | 675.51(0.03) | 2.32±0.63 | 2.98±0.86 | 0.00 | 1535.27(0.00) | 1.66±0.60 | 2.20±0.79 | 0.00 | 2.44(0.62) | 89.40 |
| Male & AD | 8 | 105 | 1.20±0.60 | 1.19±0.52 | 0.96 | 1.28(0.90) | 3.64±0.94 | 4.66±0.78 | 0.00 | 0.00(0.24) | 1.41±0.26 | 1.34±0.36 | 0.65 | 230837.34(0.16) | 1.70±0.76 | 2.81±0.69 | 0.00 | 1.43(0.95) | 1.19±0.44 | 2.26±0.87 | 0.01 | 182898.84(0.16) | 95.70 |
| Male & HCs | 25 | 78 | 1.93±2.78 | 1.34±0.66 | 0.23 | 1.13(0.90) | 4.27±0.97 | 4.35±0.88 | 0.68 | 0.09(0.34) | 1.37±0.45 | 1.21±0.27 | 0.09 | 0.29(0.75) | 2.19±0.65 | 2.62±0.73 | 0.03 | 64.49(0.17) | 1.73±0.57 | 2.25±0.75 | 0.01 | 0.65(0.82) | 82.30 |
| Female & AD | 10 | 175 | 1.68±0.31 | 1.52±0.74 | 0.63 | 0.70(0.80) | 4.35±0.65 | 5.05±1.08 | 0.13 | 0.11(0.60) | 1.55±0.27 | 1.42±0.37 | 0.36 | 205.15(0.44) | 2.15±0.44 | 3.07±0.89 | 0.01 | 0.85(0.98) | 1.40±0.28 | 2.28±0.84 | 0.01 | 1558.62(0.23) | 94.50 |
| Female & HCs | 28 | 120 | 1.51±0.69 | 1.57±1.35 | 0.78 | 16.18(0.00) | 4.48±0.72 | 4.74±0.97 | 0.31 | 0.00(0.00) | 1.43±0.35 | 1.38±0.31 | 0.56 | 9922.84(0.03) | 2.37±0.68 | 2.78±0.80 | 0.07 | 8452.25(0.00) | 1.75±0.66 | 2.09±0.69 | 0.08 | 3.00(0.63) | 86.50 |

| Table S4: Comparison of lipids between male and female (Adjusting for age) (mean±sd) | | | | | | | | | | | | | | | | | | | | | | | |
| --- | --- | --- | --- | --- | --- | --- | --- | --- | --- | --- | --- | --- | --- | --- | --- | --- | --- | --- | --- | --- | --- | --- | --- |
|  | n | | TG (mmol/L) | | | | TC (mmol/L) | | | | HDL (mmol/L) | | | | LDL (mmol/L) | | | | LDL/HDL | | | |  |
|  | Male | Female | Male | Female | P | logistics (Exp, P) | Male | Female | P | logistics (Exp, P) | Male | Female | P | logistics (Exp, P) | Male | Female | P | logistics (Exp, P) | Male | Female | P | logistics (Exp, P) | predictions correct (%) |
| overall | 216 | 333 | 1.34±1.17 | 1.55±0.99 | 0.06 | 2.05(0.02) | 4.45±0.87 | 4.86±1.02 | 0.00 | 0.53(0.40) | 1.30±0.35 | 1.41±0.34 | 0.01 | 1.13(0.92) | 2.61±0.75 | 2.88±0.86 | 0.00 | 7.25(0.03) | 2.14±0.82 | 2.14±0.79 | 0.99 | 2.77(0.03) | 65.70 |
| AD | 113 | 185 | 1.19±0.52 | 1.53±0.72 | 0.00 | 5.70(0.00) | 4.57±0.84 | 5.01±1.07 | 0.00 | 0.29(0.28) | 1.35±0.35 | 1.42±0.36 | 0.12 | 1.91(0.69) | 2.72±0.76 | 3.02±0.89 | 0.01 | 13.00(0.05) | 2.17±0.89 | 2.24±0.85 | 0.61 | 0.27(0.07) | 69.70 |
| HCs | 103 | 148 | 1.51±1.59 | 1.56±1.25 | 0.83 | 1.05(0.92) | 4.32±0.90 | 4.69±0.93 | 0.01 | 1.88(0.65) | 1.25±0.34 | 1.39±0.31 | 0.01 | 0.20(0.46) | 2.50±0.73 | 2.70±0.79 | 0.11 | 3.28(0.45) | 2.10±0.74 | 2.03±0.69 | 0.53 | 0.13(0.07) | 66.90 |
| *APOEε4+* | 71 | 116 | 1.20±0.61 | 1.43±0.61 | 0.08 | 1.14(0.83) | 4.55±0.79 | 5.17±1.14 | 0.00 | 6.43(0.29) | 1.36±0.28 | 1.44±0.39 | 0.26 | 1.51(0.88) | 2.73±0.63 | 3.16±0.95 | 0.01 | 0.12(0.31) | 2.08±0.63 | 2.32±0.89 | 0.17 | 2.32(0.53) | 66.10 |
| *APOEε4-* | 145 | 217 | 1.39±1.32 | 1.61±1.15 | 0.19 | 2.82(0.01) | 4.42±0.91 | 4.69±0.90 | 0.03 | 0.20(0.15) | 1.28±0.37 | 1.39±0.31 | 0.02 | 1.13(0.94) | 2.57±0.79 | 2.71±50.76 | 0.16 | 33.26(0.01) | 2.16±0.89 | 2.04±0.70 | 0.35 | 0.12(0.01) | 71.80 |
| *APOEε2+* | 33 | 38 | 1.75±2.43 | 1.55±0.61 | 0.71 | 0.40(0.21) | 4.11±0.99 | 4.44±0.69 | 0.19 | 22.35(0.16) | 1.38±0.41 | 1.46±0.33 | 0.42 | 0.02(0.31) | 2.07±0.70 | 2.32±0.63 | 0.21 | 0.19(0.56) | 1.59±0.59 | 1.66±0.60 | 0.72 | 0.23(0.57) | 61.70 |
| *APOEε2-* | 183 | 195 | 1.25±0.58 | 1.54±1.03 | 0.01 | 4.02(0.00) | 4.53±0.83 | 4.92±1.04 | 0.00 | 0.41(0.31) | 1.29±0.33 | 1.40±0.34 | 0.01 | 1.93(0.63) | 2.73±0.71 | 2.95±0.86 | 0.01 | 8.53(0.04) | 2.26±0.82 | 2.20±0.79 | 0.58 | 0.24(0.03) | 67.40 |
