# Supplementary material for: Effects of Sex on the Relationship Between Apolipoprotein E Gene and Serum Lipid Profiles in Alzheimer’s Disease
Source: Front Aging Neurosci. 2022 May 30;14:844066. doi: 10.3389/fnagi.2022.844066 (PMC9190463; doi:10.3389/fnagi.2022.844066)
Supplement: Supplementary Table 1 — Comparison of lipids between AD and HCs groups (Adjusting for age); Values are expressed as mean (standard deviation); AD, Alzheimer’s disease; HCs, healthy controls; APOEε3+, APOEε3 carriers; Male and APOEε3+, male APOEε3 carriers; Male and APOEε3−, male APOEε3 non-carriers; Female and APOEε3+, female APOEε3 carrier; Female and APOEε3−, female APOEε3 non-carrier. [file Table_3.docx]

| Table S1: Comparison of lipids between AD and HCs groups (Adjusting for age) (mean±sd) | | | | | | | | | | | | | | | | | | | |
| --- | --- | --- | --- | --- | --- | --- | --- | --- | --- | --- | --- | --- | --- | --- | --- | --- | --- | --- | --- |
|  | n | | TG (mmol/L) | | | | TC (mmol/L) | | | HDL (mmol/L) | | | | LDL (mmol/L) | | | LDL/HDL | | |
|  | HCs | AD | HCs | AD | P | HCs | | AD | P | HCs | AD | P | HCs | | AD | P | HCs | AD | P |
| overall | 251 | 298 | 1.54±1.39 | 1.40±0.67 | 0.73 | 4.54±0.93 | | 4.84±1.01 | 0.00 | 1.33±0.33 | 1.39±0.36 | 0.03 | 2.62±0.77 | | 2.90±0.85 | 0.00 | 2.06±0.71 | 2.21±0.86 | 0.15 |
| *APOEε3+* | 236 | 180 | 1.54±1.44 | 1.41±0.67 | 0.30 | 4.56±0.93 | | 4.83±1.00 | 0.01 | 1.34±0.33 | 1.40±0.37 | 0.06 | 2.64±0.76 | | 2.88±0.84 | 0.01 | 2.06±0.70 | 2.19±0.86 | 0.33 |
| *APOEε3-* | 15 | 18 | 1.53±1.61 | 1.25±0.68 | 0.43 | 4.23±0.92 | | 5.00±1.12 | 0.04 | 1.24±0.24 | 1.35±0.24 | 0.24 | 2.32±0.88 | | 3.18±0.78 | 0.02 | 1.95±0.84 | 2.44±0.93 | 0.17 |
| male | 103 | 113 | 1.51±1.59 | 1.19±0.52 | 0.33 | 4.32±0.90 | | 4.57±0.84 | 0.02 | 1.25±0.34 | 1.35±0.35 | 0.10 | 2.50±0.73 | | 2.72±0.76 | 0.02 | 2.10±0.74 | 2.17±0.89 | 0.39 |
| female | 148 | 185 | 1.56±1.25 | 1.53±0.72 | 0.69 | 4.69±0.93 | | 5.01±1.07 | 0.04 | 1.39±0.31 | 1.42±0.36 | 0.12 | 2.70±0.79 | | 3.02±0.89 | 0.05 | 2.03±0.69 | 2.23±0.85 | 0.42 |
| Male & *APOEε3+* | 99 | 104 | 1.49±1.62 | 1.22±1.53 | 0.48 | 4.33±0.91 | | 4.58±0.86 | 0.02 | 1.26±0.34 | 1.34±0.36 | 0.18 | 2.50±0.74 | | 2.72±0.78 | 0.04 | 2.10±0.75 | 2.19±0.92 | 0.36 |
| Male & *APOEε3-* | 4 | 9 | 1.90±0.92 | 0.74±0.15 | 0.16 | 4.19±0.80 | | 4.46±0.60 | 0.32 | 1.06±0.14 | 1.42±0.26 | 0.15 | 2.31±0.64 | | 2.67±0.65 | 0.24 | 2.20±0.57 | 1.90±0.35 | 0.85 |
| Female & *APOEε3+* | 137 | 176 | 1.58±1.30 | 1.53±0.73 | 0.72 | 4.73±0.92 | | 4.98±1.06 | 0.09 | 1.40±0.32 | 1.43±0.37 | 0.12 | 2.73±0.77 | | 2.99±0.87 | 0.04 | 2.04±0.67 | 2.20±0.82 | 0.99 |
| Female & *APOEε3-* | 11 | 9 | 1.39±0.47 | 1.62±0.68 | 0.50 | 4.25±1.01 | | 5.39±1.28 | 0.08 | 1.31±0.24 | 1.30±0.22 | 0.94 | 2.33±0.99 | | 3.54±1.12 | 0.04 | 1.86±0.94 | 2.82±1.05 | 0.10 |

| Table S2: Comparison of lipids between *APOEε3+* group and *APOEε3-* group (Adjusting for age) (mean±sd) | | | | | | | | | | | | | | | | | |
| --- | --- | --- | --- | --- | --- | --- | --- | --- | --- | --- | --- | --- | --- | --- | --- | --- | --- |
|  | n | | TG (mmol/L) | | | TC (mmol/L) | | | HDL (mmol/L) | | | LDL (mmol/L) | | | LDL/HDL | | |
|  | *APOEε3+* | *APOEε3-* | *APOEε3+* | *APOEε3-* | P | *APOEε3+* | *APOEε3-* | P | *APOEε3+* | *APOEε3-* | P | *APOEε3+* | *APOEε3-* | P | *APOEε3+* | *APOEε3-* | P |
| overall | 187 | 362 | 1.47±1.09 | 1.38±0.65 | 0.71 | 4.71±0.98 | 4.63±1.08 | 0.74 | 1.37±0.35 | 1.30±0.24 | 0.33 | 2.77±0.81 | 2.77±1.01 | 1.00 | 2.13±0.79 | 2.20±0.91 | 0.68 |
| AD | 149 | 149 | 1.41±0.67 | 1.25±0.68 | 0.52 | 4.83±1.00 | 5.00±1.12 | 0.65 | 1.40±0.37 | 1.35±0.34 | 0.57 | 3.15±0.88 | 2.88±0.84 | 0.29 | 2.19±0.86 | 2.44±0.93 | 0.35 |
| HCs | 38 | 213 | 1.54±1.44 | 1.53±0.61 | 0.93 | 4.56±0.93 | 4.23±0.92 | 0.29 | 1.34±0.33 | 1.24±0.24 | 0.35 | 2.64±0.76 | 2.32±0.88 | 0.19 | 2.06±0.70 | 1.95±0.84 | 0.61 |
| Male | 71 | 145 | 1.35±1.19 | 1.17±0.78 | 0.81 | 4.46±0.89 | 4.36±0.64 | 0.87 | 1.30±0.35 | 1.29±0.28 | 0.89 | 2.62±0.77 | 2.54±0.52 | 0.86 | 2.14±0.84 | 2.01±0.43 | 0.73 |
| Female | 116 | 217 | 1.55±1.02 | 1.50±0.57 | 0.78 | 4.87±1.00 | 4.78±1.25 | 0.80 | 1.42±0.35 | 1.30±0.22 | 0.18 | 2.87±0.83 | 2.90±1.19 | 0.81 | 2.13±0.76 | 2.31±1.08 | 0.30 |
